# The severity of ischemic stroke and risk of all-cause mortality in patients with atrial fibrillation on different oral anticoagulant treatments admitted to the emergency department

**DOI:** 10.1007/s11239-025-03095-1

**Published:** 2025-04-05

**Authors:** Tommasa Vicario, Danilo Menichelli, Alfredo Paolo Mascolo, Marina Diomedi, Sara Cerretti, Francesco Marconi, Pasquale Pignatelli, Carla Paganelli, Daniele Pastori

**Affiliations:** 1 Emergency Department, Policlinico Tor Vergata Hospital, Rome, Italy; 2 Department of General Surgery and Surgical Specialties “Paride Stefanini”, Sapienza University of Rome, Rome, Italy; 3 Stroke Center, Department of Systems Medicine, University Hospital of Rome ‘Tor Vergata’, Rome, Italy; 4 Emergency Department, M.G. Vannini Hospital, “Istituto delle Figlie di San Camillo”, Rome, Italy; 5 Department of Clinical Internal, Anesthesiological and Cardiovascular Sciences, Sapienza University of Rome, Viale del Policlinico 155, Rome, 00185 Italy; 6 IRCCS Neuromed, Località Camerelle, Pozzilli, IS 86077 Italy

**Keywords:** Atrial fibrillation, Ischemic stroke, IS, Severity, NIHSS, DOAC, VKA

## Abstract

Although direct oral anticoagulants (DOACs) are non-inferior to Vitamin K antagonists (VKA) in preventing ischemic stroke (IS) in atrial fibrillation (AF) patients, there are limited data regarding stroke severity and prognosis of patients admitted with IS during DOAC treatment. We performed a single center retrospective study including patients with AF on oral anticoagulants admitted to the Emergency Department for IS were included. The primary endpoint was to analyse the severity of stroke evaluated through NIHSS scale according to anticoagulant therapy. The secondary endpoint was 3-month all-cause mortality. A total of 106 AF patients were included, with a mean age of 81.3 ± 7.5 years. Overall, 54.7% were women and 61.3% on DOAC. The AF patients on DOAC were older, with no other clinical differences. Median NIHSS was 12 (Interquartile Range [IQR] 5–19). At multivariable logistic regression analysis DOAC use (compared to warfarin) was associated with lower risk of moderate-severe/severe stroke (NIHSS ≥ 16) (Odds Ratio [OR] 0.355, 95% confidence interval [95% CI] 0.127–0.995). Mechanical thrombectomy was strongly associated with higher severity of stroke (OR 6.113, 95%CI 2.186–17.099). During follow-up, 42 patients died. DOAC use inversely correlated with mortality risk (OR 0.323, 95%CI 0.127–0.822) after adjusting for CHA_2_DS_2_-VASc, time to hospital admission from symptom onset and type of acute treatment. In conclusion, in our contemporary real-world population, patients on DOACs treatment admitted for IS had better outcomes in terms of stroke severity and all-cause mortality compared with patients on VKAs.

**Graphical Abstract:**

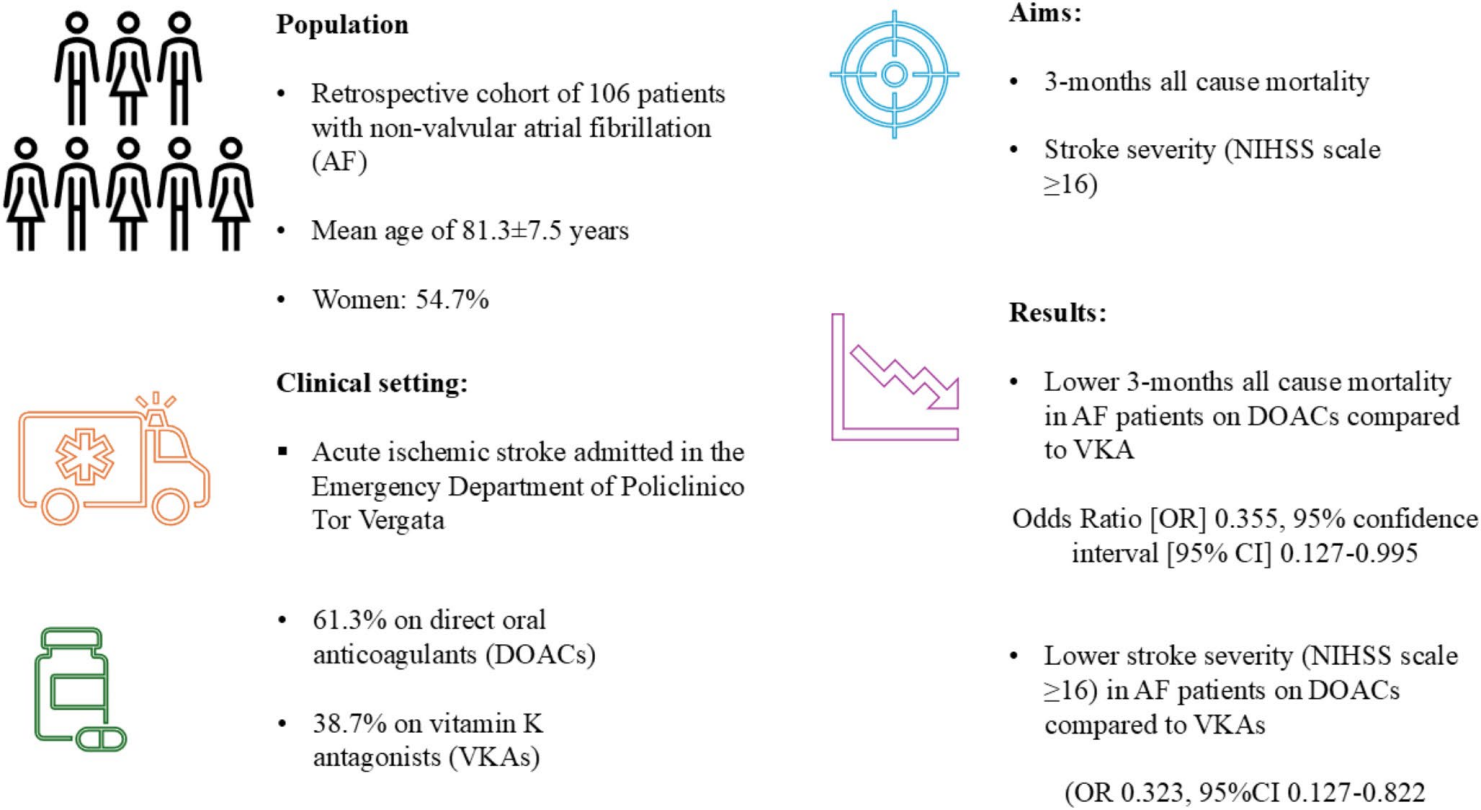

## Key points

Direct oral anticoagulants (DOACs) were associated with a lower severity of ischemic stroke (IS) in patients with non-valvular atrial fibrillation (AF).

DOACs were associated with a lower 3-months all-cause mortality risk in patients with AF.

The use of mechanical thrombectomy was strongly associated with a higher severity of stroke in AF patients.

Switching to DOAC in AF patients on vitamin K antagonists (VKA) suffering from an IS may provide a survival benefit.

## Introduction

Atrial fibrillation (AF) is the most sustained cardiac arrythmia and currently the estimated prevalence in adult population is between 2% and 4% [1]. Owing to extended longevity in general population and improvement of screening tool, a 2.3-fold rise in incidence is expected [2]. AF-related outcomes are represented by stroke, heart failure, sudden death, and cardiovascular morbidity. Studies show that 20–30% of all ischaemic stroke are due to AF [3] and acute ischemic stroke (IS) in the setting of AF is more disabling and carries an increased risk of mortality than patients without this arrythmia [4]. AF increases stroke risk of five-fold and this risk is not homogeneous, depending on the presence of specific stroke risk factors/modifiers.

Stroke prevention therapies are represented by Vitamin K antagonists (VKA) or Direct Oral Anticoagulants (DOACs). Registration trials have demonstrated that DOACs are non-inferior to VKA in the prevention of systemic embolism and IS in patients affected by nonvalvular AF and have a better safety profile as regard to overall bleedings and especially intracranial hemorrhages [5]. Furthermore, they were associated with a significant 10% reduction in all-cause mortality [6] and with a reduction of major cardiac events [7], so that they are recommended as first-line choice by current ESC Guidelines for stroke prevention in AF [5].

Despite the reduction of risk of stroke after the introduction of DOACs, few data on the severity of IS according to different anticoagulant treatments are available. Furthermore, real life studies provided discordant data [8, 9]. Also, data on functional outcome after the acute event are sparce [10, 11].

Our aim was to investigate stroke severity on admission to the Emergency Department and short-term all-cause mortality in a cohort of patients with AF according to different OAC. In particular, the primary endpoint of the study is to compare the prevalence of moderate-severe/severe stroke according to National Institutes of Health Stroke Scale (NIHSS) in patients treated with DOACs or VKAs. In addition, we estimated the all-cause mortality risk at 3 months.

## Methods

### Study design and participants

This monocentric retrospective trial enrolls patients with AF pretreated with OAC who experience IS and that were admitted to the Emergency Department of Policlinico Tor Vergata between 2019 and 05 and 2022-07. Patients were included in this study if they had IS according to the World Health Organization criteria [12] (central nervous system infarction is brain, spinal cord, or retinal cell death attributable to ischemia, based on (1) pathological, imaging, or other objective evidence of cerebral, spinal cord, or retinal focal ischemic injury in a defined vascular distribution; or (2) clinical evidence of cerebral, spinal cord, or retinal focal ischemic injury based on symptoms persisting ≥ 24 h or until death, and other etiologies excluded), if they had history of AF and assumed anticoagulant therapy either with DOACs or VKAs with international normalized ratio (INR) range established between 2-0-3.0. Patients with AF and mechanical prosthetic heart valve replacement, active cancer, preceding antithrombotic therapy other than anticoagulant, stroke mimics and non-cerebral ischemic events were excluded.

The study was carried out according to the principles of the Declaration of Helsinki and approved by the Sapienza University of Rome Ethics committee (Prot. 0405/2022).

We grouped patients according to the strata of OAC prescription (VKA, DOAC) at the admission to hospital. We collected data regarding stroke severity (NIHSS), pre-stroke dependence: modified Rankin Scale (mRS), time of onset symptoms or information about last time the patient was seen well, INR value and the time of last DOAC dose assumption. This specific assessment was performed by a neurovascular consultant who, on the basis of these data and radiological findings, prescribed the most appropriate treatment according to international current Guidelines on the management of IS, using thrombolysis with recombinant Tissue Plasminogen Activator (r-TPA) or mechanical thrombectomy, both or none [13, 14]. **All patients**,** independently from the type of oral anticoagulants taking at admission**,** were stopped and re-started on oral anticoagulants according to international guidelines** [15, 16].

We also collected information on anthropometric variables and past medical history including age, sex, vital signs, estimated time of symptoms occurrence, data on past medical history, especially, cardiovascular risk factors, pattern of AF, pretreatment as antihypertensive, lipid lowering medication, antiarrhythmic drug, anticoagulants, laboratory tests (glucose, creatinine, blood red cells, platelets, INR, cholesterol, troponin and type of treatment (intravenous thrombolysis [IVT] and endovascular therapy [EVT] or no treatment. Information on the length of hospital stay, hemorrhagic transformation was also recorded.

### Outcomes

The primary endpoint of this study was to assess stroke severity on admission, which was evaluated through NIHSS scale by neurologists. Stroke severity was categorized as follows: no stroke symptoms, 0; minor stroke, 1–4; moderate stroke, 5–15; moderate to severe stroke, 16–20; and severe stroke, 21–42 according to recent evidence [17].

The secondary outcome was 3 months all-cause mortality.

### Statistical analysis

Categorical variables are reported as counts (percentage). Pearson χ2 test was used to compare proportions. Continuous variables are expressed as mean and standard deviation or median and interquartile range (IQR) as appropriate according to Kolmogorov-Smirnov test to evaluate the distribution of each variable. Student t-test and Mann-Whitney U test were used to compare means and median, respectively.

A first descriptive analysis of clinical characteristics according to OAC used, stroke severity and all-cause of death was performed. We used univariable logistic regression analysis to calculate the relative odds ratio (OR) and the 95% confidence interval (95% CI) to estimate the association of DOAC use with moderate-severe stroke and all-cause mortality. Then, we performed 4 models using the multivariable logistic regression analysis. The first model evaluated the association of DOAC use with moderate-severe stroke at presentation and all-cause mortality adjusting for sex and age, while the second one adjusting for CHA_2_DS_2_-VASc. Then we performed a further model adjusting for CHA_2_DS_2_-VASc and IS treatment, and, finally, a model adjusted for CHA_2_DS_2_-VASc, IS treatment and time to admission to hospital from onset of symptoms.

All p values < 0.05 were considered statistically significant. Statistical analysis was performed with IBM SPSS 25.0 software.

## Results

### Patients’ characteristics

We included 65 patients on DOAC (81.5% on Factor Xa inhibitors) and 41 on VKA. Baseline characteristics of patients according to OAC status before the acute event are reported in Table 1. Mean age was 81.34 ± 7.5 and 77.4% were affected by hypertension, 26.4% by diabetes and the mean CHA_2_DS_2_-VASc was 3.74 ± 1.0. **No difference was found about age**,** previous cardiovascular and cerebrovascular disease and cardiovascular risk factors between patients on DOAC or VKA.** Furthermore, no significant difference was found on haemoglobin, platelet count, creatinine and blood glucose among groups (Table 1).

**Table 1:** Baseline characteristics comparing patients on treatment with VKA or DOAC

	Total Cohort (*n*:106)	VKA (*n*:41)	DOAC (*n*:65)	*p*-value
Age (years)	81.34 ± 7.5	81.2 ± 8.6	81.6 ± 6.7	**0.813**
Women (%)	54.7	53.7	55.4	1.000
Hypertension (%)	77.4	70.7	81.5	0.236
Diabetes (%)	26.4	24.4	27.7	0.822
Previous stroke (%)	17.1	15.0	18.5	0.792
Previous cardiovascular disease (%)	25.5	19.5	29.2	0.361
Heart failure (%)	14.2	14.6	13.8	1.000
CHA_2_DS_2_-VASc score	3.74 ± 1.0	3.6 ± 1.0	3.9 ± 1.0	0.106
NIHSS median (IQR)	12 (5–19)	16 (8–20)	10 (5–16)	0.032
Systolic Blood pressure (mmHg)	149.8 ± 30.0	144.1 ± 24.2	153.8 ± 32.9	0.123
Diastolic Blood pressure (mmHg)	82.0 ± 15.8	79.2 ± 14.4	83.9 ± 16.6	0.166
Time to admission (hours) (IQR)	2.0 (1.0–5.0)	2.0 (1.0–4.0)	3.0 (1.0-5.8)	0.568
Laboratory Test				
International normalized ratio	-	1.75 ± 0.7	-	-
Haemoglobin (g/dl)	12.7 ± 1.9	12.5 ± 1.9	12.8 ± 2.0	0.369
Platelet count (x 109/mmc)	235.2 ± 89.4	231.5 ± 98.3	237.8 ± 83	0.746
Creatinine (mg/dl)	1.2 ± 0.6	1.3 ± 0.9	1.1 ± 0.4	0.105
Blood glucose (mg/dl)	139.4 ± 60.0	144.4 ± 63.3	135.9 ± 57.8	0.514
Therapy				
Statins (%)	33.3	33.3	33.3	1.000
Beta blockers (%)	64.7	69.2	61.9	0.525
ACE-I/ARBs (%)	59.8	53.8	63.5	0.407
CCB (%)	17.6	23.1	14.3	0.292
Antiarrhythmic drugs (%)	65.3	64.1	66.1	0.834
Treatment of stroke				
None (%)	46.2	31.7	55.4	0.065
Thrombolysis (%)	9.4	9.8	9.2	
Mechanical thrombectomy (%)	35.8	43.9	30.8	
Mechanical thrombectomy + thrombolysis (%)	8.5	14.6	4.6	

ACE-I/ARBs: angiotensin converting enzyme/angiotensin receptor blockers, CCB: calcium channel blockers, DOAC: direct oral anticoagulant, IQR: interquartile range, NIHSS: national institutes of health stroke scale, VKA: vitamin K antagonist

Stroke severity on admission was higher in VKA patients compared to DOAC (median 16.0, interquartile range [IQR] 8.0–20.0, and median 10.0, IQR 5.0–16.0, respectively, *p* = 0.032). No difference on time to admission from onset of IS symptoms (Table 1). Patients treated with VKA were more likely to be treated with thrombectomy compared to DOACs ones (58.5% vs. 35.4%, *p* = 0.019).

We also evaluated patients’ characteristics according to stroke severity on admission. We defined a minor-mild stroke class (NIHSS 0–15) and a moderate/severe-severe one (NIHSS 16–42). In our population, patients with severe stroke were 37/106, were more often female and were less treated with DOAC (Table 2). No difference was found about previous cardiovascular and cerebrovascular disease and common cardiovascular risk factors as hypertension and diabetes. Furthermore, no significant difference was found on haemoglobin, platelet count, creatinine and blood glucose among groups (Table 2).

**Table 2:** Characteristics of patients according to stroke severity

	Total Cohort (*n*:106)	NIHSS < 16 (*n*:69)	NIHSS ≥ 16 (*n*:37)	*p*-value
Age (years)	81.34 ± 7.5	80.9 ± 6.7	82.4 ± 8.5	0.336
Women (%)	54.7	44.9	73.0	0.008
Hypertension (%)	77.4	79.7	73.0	0.470
Diabetes (%)	26.4	30.4	18.9	0.251
Previous stroke (%)	17.1	17.4	16.7	1.000
Previous cardiovascular disease (%)	25.5	30.4	16.2	0.160
Heart failure (%)	14.2	11.6	18.9	0.382
DOAC (%)	61.3	72.5	40.5	0.002
CHA_2_DS_2_-VASc score	3.74 ± 1.0	3.72 ± 1.0	3.76 ± 0.9	0.872
Systolic Blood pressure (mmHg)	149.8 ± 29.9	149.4 ± 31.2	150.7 ± 27.8	0.839
Diastolic Blood pressure (mmHg)	82.0 ± 15.8	79.9 ± 15.0	85.8 ± 16.8	0.085
Time to admission (hours) (IQR)	2.0 (1.0–5.0)	3.0 (1.5–6.5)	2.0 (1.0-3.3)	0.046
Laboratory Test				
International normalized ratio	1.5 ± 0.6	1.4 ± 0.6	1.6 ± 0.6	0.339
Haemoglobin (g/dl)	12.7 ± 1.9	12.3 ± 2.1	12.9 ± 1.8	0.274
Platelet count (x 109/mmc)	235.2 ± 89.4	233.2 ± 83.3	236.4 ± 93.1	0.688
Creatinine (mg/dl)	1.2 ± 0.6	1.2 ± 0.6	1.2 ± 0.7	0.743
Blood glucose (mg/dl)	139.4 ± 60.0	142.4 ± 59.9	137.8 ± 60.4	0.665
Therapy				
Statins (%)	33.3	38.2	23.5	0.182
Beta blockers (%)	64.7	63.2	67.6	0.826
ACE-I/ARBs (%)	59.8	61.8	55.9	0.669
CCB (%)	17.6	14.7	23.5	0.283
Antiarrhythmic drugs (%)	65.3	67.2	61.8	0.660
Treatment of stroke				
None (%)	46.2	59.4	21.6	< 0.001
Thrombolysis (%)	9.4	11.6	5.4	
Mechanical thrombectomy (%)	35.8	23.3	59.5	
Mechanical thrombectomy + thrombolysis (%)	8.5	5.8	13.5	

ACE-I/ARBs: angiotensin converting enzyme/angiotensin receptor blockers, CCB: calcium channel blockers, DOAC: direct oral anticoagulant, IQR: interquartile range, NIHSS: national institutes of health stroke scale

Regarding acute treatment, patients with IS and a NIHSS ≥ 16 were more commonly treated with mechanical thrombectomy or with combined therapy of pharmacological thrombolysis and mechanical thrombectomy compared to patients with NIHSS < 16 (Table 2).

### Anticoagulant treatments and severity of stroke

We evaluated also the risk of moderate/severe stroke in DOAC users compared to VKA. At univariable logistic regression analysis (Table 3, Panel A, Model A), DOACs has been associated to a lower risk to develop a moderate-severe/severe stroke (NIHSS ≥ 16) (Odds Ratio [OR] 0.326, 95% confidence interval [CI] 0.182–0.584, *p* < 0.001). This result was confirmed by multivariable logistic regression adjusted for age and sex (Table 3, Panel A, Model B) (OR 0.255, 95%CI 0.105–0.169, *p* = 0.003), CHA_2_DS_2_-VASc (Table 3, Panel A, Model C) (OR 0.278, 95%CI 0.118–0.652, *p* = 0.003). This association was confirmed also adjusting for CHA_2_DS_2_-VASc and stroke treatment (Table 3, Panel A, Model D) (OR 0.291, 95%CI 0.112–0.757, *p* = 0.011) and for CHA_2_DS_2_-VASc, stroke treatment and time to admission in hospital from symptoms onset (Table 3, Panel A, Model E) (OR 0.355, 95%CI 0.127–0.995, *p* = 0.049). Mechanical thrombectomy, but no time to admission in hospital, use was strongly associated with severity of stroke (OR 6.113, 95%CI 2.186–17.099, *p* = 0.001).

**Table 3:** Association between the use of oral anticoagulant and risk of moderate-severe/severe stroke (Panel A) and all-cause mortality (Panel B)

	Model A^*^			Model B^**^			Model C^***^			Model D^+^				Model E^++^			
	OR	95%CI	*p*-value	OR	95%CI	*p*-value	OR	95%CI	*p*-value	OR	95%CI		*p*-value	OR	95%CI		*p*-value
A. *Stroke with NIHSS*$\:\ge\:16$																	
**DOAC use**	0.326	0.182–0.584	< 0.001	0.255	0.105–0.619	0.003	0.278	0.118–0.652	0.003	0.291		0.112–0.757	0.011	0.355		0.127–0.995	0.049
B. *All-cause Mortality*																	
**DOAC use**	0.455	0.204–1.015	0.054	0.455	0.203–1.020	0.056	0.463	0.205–1.048	0.065	0.441		0.187–1.043	0.062	0.323		0.127–0.822	0.018

DOAC: direct oral anticoagulants, OR: Odds Ratio, 95%CI: 95% confidence interval

*Model A: univariable. **Model B: adjusted for age and sex. ***Model C: adjusted for CHA_2_DS_2_-VASc, ^+^Model D: adjusted for CHA_2_DS_2_-VASc, stroke treatment, ^++^Model E: adjusted for CHA_2_DS_2_-VASc, stroke treatment and time to admission

### Anticoagulant treatments and all-cause of death

Patients’ characteristics according to death status were also evaluated. In our population, 42 patients died. No difference was found about previous cardiovascular and cerebrovascular disease, cardiovascular risk factors, NIHSS scale at admission, time to admission or concomitant treatments (Table 4). No differences between IS acute treatment were observed (Table 4). Furthermore, no significant difference was found on haemoglobin, platelet count, creatinine and blood glucose among groups (Table 4).

**Table 4:** Baseline characteristics comparing to living status

	Total Cohort (*n*:106)	Alive (*n*: 64)	Dead (*n*: 42)	*p*-value
Age (years)	81.4 ± 7.4	81.6 ± 7.2	81.2 ± 7.7	0.764
Women (%)	54.7	57.8	50.0	0.550
Hypertension (%)	77.4	76.6	78.6	1.000
Diabetes (%)	26.4	32.8	16.7	0.075
Previous stroke (%)	17.1	14.3	21.4	0.430
Previous cardiovascular disease (%)	25.5	23.4	28.6	0.650
Heart failure (%)	14.2	10.9	19.0	0.266
DOAC (%)	61.3	68.8	50.0	0.067
CHA_2_DS_2_-VASc score	3.7 ± 1.0	3.8 ± 1.0	3.7 ± 1.0	0.554
NIHSS median (IQR)	12.0 (5–19)	11.5 (4–18)	14.5 (8-19.75)	0.060
NIHSS $\:\ge\:16$(%)	34.9	29.7	42.9	0.212
Time to admission (hours) (IQR)	2.0 (1.0–5.0)	2.0 (1.0–6.0)	2.0 (1.5-4.0)	0.914
Systolic Blood pressure (mmHg)	149.8 ± 29.9	150.2 ± 30.6	149.3 ± 29.2	0.894
Diastolic Blood pressure (mmHg)	82.0 ± 15.8	82.0 ± 16.5	81.9 ± 15.0	0.966
Time to admission (hours) (IQR)	2.0 (1.0–5.0)	2.0 (1.0–6.0)	2.0 (1.5-4.0)	0.861
Laboratory Test				
International normalized ratio	1.5 ± 0.6	1.4 ± 0.6	1.6 ± 0.6	0.304
Haemoglobin (g/dl)	12.7 ± 1.9	12.7 ± 1.9	12.6 ± 1.9	0.453
Platelet count (x 109/mmc)	235.2 ± 89.4	246.5 ± 102.0	227.7 ± 102.1	0.240
Creatinine (mg/dl)	1.2 ± 0.6	1.1 ± 0.5	1.3 ± 0.8	0.075
Blood glucose (mg/dl)	139.4 ± 60.0	134.2 ± 51.3	147.7 ± 71.6	0.481
Therapy				
Statins (%)	33.3	33.3	33.3	1.000
Beta blockers (%)	64.7	60.0	71.4	0.294
ACE-I/ARBs (%)	59.8	58.3	61.9	0.838
CCB (%)	17.6	11.7	26.2	0.069
Antiarrhythmic drugs (%)	65.3	60.0	73.2	0.205
Treatment of stroke				
None (%)	48.4	42.9	46.2	0.333
Thrombolysis (%)	10.9	7.1	9.4	
Mechanical thrombectomy (%)	29.7	45.2	35.8	
Mechanical thrombectomy + thrombolysis (%)	10.9	4.8	8.5	

ACE-I/ARBs: angiotensin converting enzyme/angiotensin receptor blockers, CCB: calcium channel blockers, DOAC: direct oral anticoagulant, IQR: interquartile range, NIHSS: national institutes of health stroke scale

At univariable logistic regression analysis (Table 3, Panel A, Model A) no association was observed between patients treated with DOACs and VKAs (OR 0.455, 95%CI 0.204–1.015, *p* = 0.054). This finding was confirmed at multivariable logistic regression adjusting for age and sex (Table 3, Panel B, Model B), CHA_2_DS_2_-VASc (Table 3, Panel B, Model C) and also stroke treatment (Table 3, Panel B, Model D). However, in the Model E adjusted for CHA_2_DS_2_-VASc, stroke treatment and time to admission in hospital from symptoms onset (Table 3, Panel B), DOAC use was associated with lower risk of all-cause mortality (OR 0.323. 95%CI 0.127–0.822, *p* = 0.018).

## Discussion

This real-world comparison of patients admitted to the Emergency Department of a University Hospital for acute IS while on treatment either with VKA or DOAC showed that stroke severity at hospital admission was lower in patients on DOAC. In addition, we found patients treated with DOACs had a lower mortality rate compared to VKA at 3 months.

Furthermore, we found that thrombectomy was associated with higher stroke severity with similar results at 3 months all-cause mortality compared to fibrinolysis. The use of thrombectomy instead of thrombolysis may be explained by the indication to use thrombectomy in patients with more severe stroke, such as those with large vessel occlusion that could be associated with more severe symptoms of stroke [18]. Furthermore, we found that thrombectomy treatment was more frequently performed in the VKAs group. This may be explained by current recommendation [13] that excludes thrombolysis, patients with INR values > 1.7, making thrombectomy the preferred choice in this subclass of patients. Of note, no significant differences were found on INR between high and low stroke severity in our cohort, although the median INR was under normal range in both groups.

Previous studies compared patients with IS on anticoagulant treatment to no treatment showing a benefit of anticoagulants, especially if in therapeutic range, in the prevention of mortality or stroke severity. A retrospective cohort study performed on 3,669 patients with IS showed that the use of DOAC or phenprocoumon with INR ≥ 2 was associated with a lower risk of stroke severity at admission compared to AF patients without not taking thromboprophylaxis [19]; no data about all-cause mortality were reported in this study [19]. In addition, a retrospective study performed on 330 AF patients with hospitalization for IS showed that anticoagulation was associated with a lower IS severity compared with no treatment. However, this study did not compare type of oral anticoagulants [20].

Finally, a recent systematic review and meta-analysis [21], that examined 9,493 patients with AF and IS found that patients with non-therapeutical VKA presented with more severe stroke [21]. **This meta-analysis included only 6 studies**,** 2 of them did not included DOAC and did not directly compare DOAC and VKA** [22, 23], **while**,** another one did not included VKA arm and compared only no treatment or DOAC evaluating NIHSS at admission** [24]. **All studies had as outcomes the NIHSS scale at admission**,** but no data were reported on all-cause mortality**,** that as showed by our findings**,** may be lower in patients taking DOAC compared to VKA.**

Comparing different anticoagulants, our findings were coherent with a previous study [25] that enrolled 156 patients with IS treated with VKA or DOAC. However, in this study no difference about stroke severity was observed between DOAC and VKA at baseline. The cohort of this study had similar age, proportions of comorbidities and treatment strategy compared to our cohort [25]. No data about INR values and time to admission were reported in this study. For this reason, it could not be established if the similar stroke severity between VKA and DOAC group of previous study could be ascribed to these factors [25].

Furthermore, a study performed on 2,173 AF patients [26] of whom 628 on warfarin, 272 on DOACs, 429 on antiplatelets alone, and 844 without therapy showed that DOAC use was associated with smaller ischemic brain lesions and inversely associated with moderate to severe stroke (OR 0.56, 95%CI 0.40–0.78), while patients on VKA or antiplatelet showed similar stroke severity compared to no treatment [26].

Although DOACs seems to reduce the severity of stroke, this clinical condition may be a potential challenge for the clinician due to the lack of evidence-based recommendations about the switch from well-conducted therapy with VKA to DOAC or changing DOAC after the occurrence of an IS [5].

Our study also evaluated mortality risk at 3 months after IS. We found a 68% relative risk reduction of mortality in patients treated with DOACs. In the previously mentioned study [25], the use of VKA was associated with higher risk of all-cause mortality [25]. This finding suggests that the use of DOAC may have beneficial effect on short-term outcomes in AF patients after an IS.

### Clinical implications

Our study included a very high-risk cohort of patients with AF and IS. While comorbidities do not seem to be useful to identify patients with more severe stroke, the use of DOAC and treatment in the acute phase seems to be able to modify the prognosis of these patients. Indeed, home treatment with DOAC before admission was associated with a lower severity of stroke, as well as thrombectomy associated with moderate-severe stroke.

Other factors not analysed in the present study may be responsible for the severity of stroke, such as, left atrial enlargement, AF pattern, and presence of peripheral artery disease (i.e. carotid atherosclerosis).

Furthermore, it may be of interest to analyse the risk of long-term mortality and functional outcomes, such as disability or quality of life, according to the severity of stroke and anticoagulant treatment.

Finally, despite the positive association between DOAC and lower mortality, the management of the post-acute phase after an AIS is still uncertain. Thus, current European guidelines [27] report no solid evidence on the benefit of switching between different anticoagulant regimens after an acute ischemic event, and a recent clinical trial [28] shows no benefit in reducing thromboembolic risk in switching from VKA to DOAC in a cohort of elderly with good adherence to VKA.

### Limitations

An intrinsic limitation is related to the retrospective observational design of the study that is limited by the presence of residual potential confounders such as the duration of anticoagulation therapy or adherence to prescribed anticoagulants. Furthermore, our cohort had a relatively low number of patients, and for this reason, a further larger cohort study may help to confirm our results across diverse populations. In addition, we do not have data on adherence to DOAC treatment before admission. Lastly, no data about time in therapeutic range in patients treated with VKA was available, but we collected data on INR values at the moment of the clinical presentation of the AIS, that is currently used as the parameter to decide regarding in-hospital management. Furthermore, our enrollment took more time than expected as the study was conducted during the COVID-19 pandemic, with all difficulties related to the care of patients with IS [29]. Finally, these findings should be confirmed ideally with the assessment of DOAC drug activity level.

## Conclusions

Stroke severity at hospital admission seems to be inversely associated with DOAC use at home along with lower all-cause mortality at 3 months compared to VKA. **However**,** the net clinical benefit of different anticoagulants after an AIS requires further research.**

## Data Availability

The data that support the findings of this study are not openly available due to reasons of sensitivity and are available from the corresponding author upon reasonable request.
